# Radial Potential Energy Functions of Linear Halogen-Bonded Complexes YX···ClF (YX = FB, OC, SC, N_2_) and the Effects of Substituting X by Second-Row Analogues: Mulliken Inner and Outer Complexes

**DOI:** 10.1021/acs.jpca.2c01205

**Published:** 2022-04-15

**Authors:** J. Grant Hill, Anthony C. Legon

**Affiliations:** † Department of Chemistry, University of Sheffield, Sheffield S3 7HF, U.K..; ‡ School of Chemistry, University of Bristol, Cantock’s Close, Bristol BS8 1TS, U.K.

## Abstract

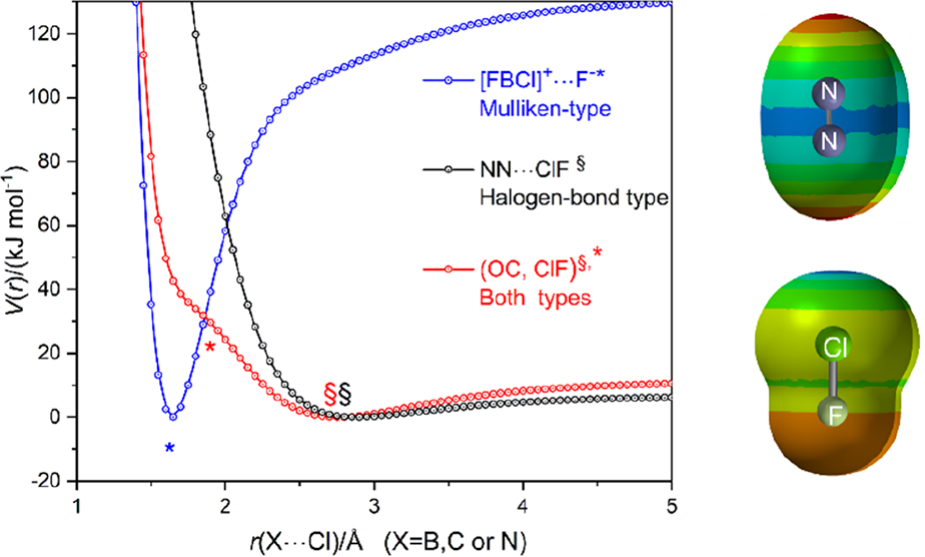

Energies of linear, halogen-bonded complexes in the isoelectronic series YX···ClF (YX = FB, OC, or N_2_) are calculated at several levels of theory as a function of the intermolecular distance *r*(X···Cl) to yield radial potential energy functions. When YX = OC, a secondary minimum is observed corresponding to lengthened and shortened distances *r*(ClF) and *r*(CCl), respectively, relative to the primary minimum, suggesting a significant contribution from the Mulliken inner complex structure [O=C–Cl]^+^···F^–^. A conventional weak, halogen-bond complex OC···ClF occurs at the primary minimum. For YX = FB, the primary minimum corresponds to the inner complex [F=B–Cl]^+^···F^–^, while the outer complex FB···ClF is at the secondary minimum. The effects on the potential energy function of systematic substitution of Y and X by second-row congeners and of reversing the order of X and Y are also investigated. Symmetry-adapted perturbation theory and natural population analyses are applied to further understand the nature of the various halogen-bond interactions.

## 1 Introduction

In a recent publication concerned with the calculation of radial potential energy functions of known linear and other axially symmetric halogen-bonded complexes B···ClF formed by chlorine monofluoride,^1^ it was found that when the Lewis base B is CO, the function contains two minima, but in the other cases only one minimum was present. The potential energy curve when the Lewis base was carbon monoxide displayed evidence of not only the expected minimum corresponding to the conventional halogen-bonded species OC···Cl, as observed experimentally,^2^ but also a secondary minimum at (*r*–*r*_e_) ≈ −1.0 Å. The C-to-Cl distance is therefore approximately 1 Å shorter than in the conventional halogen-bonded isomer OC···ClF. Moreover, the distance *r*(Cl–F) was significantly increased. An explanation of this observation is that, as the Cl atom approaches the C atom along the intermolecular axis more closely than the distance in the conventional halogen-bonded species OC···ClF, there is a chemical interaction of C and Cl which leads to partial C–Cl covalent-bond formation. This is a particular example of Mulliken’s general classification of complexes, which is based on charge transfer between an electron donor D and an electron acceptor XA.^3^ A typical halogen-bonding interaction that is almost entirely electrostatic in nature is usually signified as D···XA and corresponds to a Mulliken “outer complex”. Inner complexes are more strongly bound and may be written in the form [D–X]^+^···A^–^. Recent examples of Mulliken inner complexes are those involving the interaction of PH_3_ with ClF^4,5^ and phosphabenzene and ClF.^6^

The purpose of the present article is to investigate the observations reported in ref (1) for OC···ClF in some detail and to answer the following questions:

(a) Is the presence and position of the secondary minimum in the radial potential curve of the complex OC···ClF independent of the level of theory at which the curve is calculated?
(b) What is the electronic structure of the complex at the secondary minimum?
(c) What is the effect on the radial potential energy function when O in CO is substituted by the second-row chalcogen atom S to form the analogous complex SC···ClF?
(d) Does the secondary minimum observed for the (OC,ClF) complex occur in the related complex OSi···ClF in which C is replaced by the second row, group 14 atom Si?
(e) How does CO differ from CS and SiO in halogen-bond formation with ClF?
(f) What happens when the isoelectronic series FB···ClF, OC···ClF, and N_2_···ClF is similarly examined?

In what follows, we attempt to answer these questions by calculating the radial potential energy functions of the various B···ClF complexes using several different levels of theory and analyzing the electronic structure and nature of the interactions at the minima located on these potential energy functions.

## 2 Theoretical Methods

Relaxed potential energy scans were carried out, in which the B···Cl distance is fixed, all atoms are constrained to be collinear, and all other internal coordinates are optimized. The explicitly correlated coupled cluster CCSD(T)-F12c method [also known as CCSD(T)(F12*)]^7^ in the Molpro system of ab initio programs^8,9^ was employed. The triple-zeta correlation-consistent basis set designed specifically for use in explicitly correlated calculations, cc-pVTZ-F12,^10^ was used for all atoms, along with the aug-cc-pVTZ/MP2Fit,^11^ aug-cc-pVTZ/JKFit,^12^ and cc-pVTZ-F12/OptRI auxiliary basis sets.^13^ The geminal Slater exponent was set to 1.0 *a*_0_^–1^. To investigate the sensitivity of the relaxed scans to basis set size, some calculations were also carried out with the double-zeta cc-pVDZ-F12 basis set, along with the equivalent auxiliary basis sets.

Density functional theory calculations were carried out with the Gaussian 16 package,^14^ using two exchange–correlation functionals: M06-2X^15^ and ωB97X-D.^16^ In both cases the correlation-consistent aug-cc-pV(T+d)Z basis sets were used,^17−19^ where +d indicates that additional “tight” functions were included for second-row atoms. An ultrafine integration grid (99 radial shells and 590 angular points per shell) was also used.

Symmetry-adapted perturbation theory (SAPT) calculations were carried out to decompose the interaction energy of a complex into electrostatic, exchange, induction, and dispersion components at the SAPT2+(3)(CCD)δMP2/aug-cc-pV(T+d)Z level.^20−22^ A SAPT charge-transfer analysis^23^ was also carried out at the SAPT2+(3)(CCD)/aug-cc-pV(T+d)Z level, and all SAPT calculations were performed with the Psi4 V1.3.1 program.^24^ For brevity, SAPT2+(3)(CCD)δMP2/aug-cc-pV(T+d)Z will be referred to as SAPT herein. Natural population analysis (NPA) at the local minima used the NBO6 program^25^ interfaced to Molpro, with the MP2/aug-cc-pV(T+d)Z density. Molecular electrostatic potential maps (MESPs) were obtained at the M06-2X/6-311++G** level^26^ in the SPARTAN package,^27^ with an isodensity surface of 0.001 e bohr^–3^.

To ensure the SAPT results are reliable, the total SAPT interaction energy (*E*_I_), defined as the difference in energy between the interacting complex and its “monomers” frozen in the geometries they adopt in the interacting complex, is compared with the CCSD(T)-F12c/cc-pVTZ-F12 analogue. As SAPT is inherently free of basis set superposition errors (BSSEs), the coupled cluster interaction energies included the counterpoise correction.^28^ The magnitude of the counterpoise correction is small at the CCSD(T)-F12c/cc-pVTZ-F12 level, with an average value of 0.60 kJ mol^–1^ for the complexes under consideration.

## 3 Results

### 3.1 Evidence that the Secondary Minimum in the Radial Potential Energy Function of OC···ClF is Independent of the Method of Calculation

**Figure 1:**
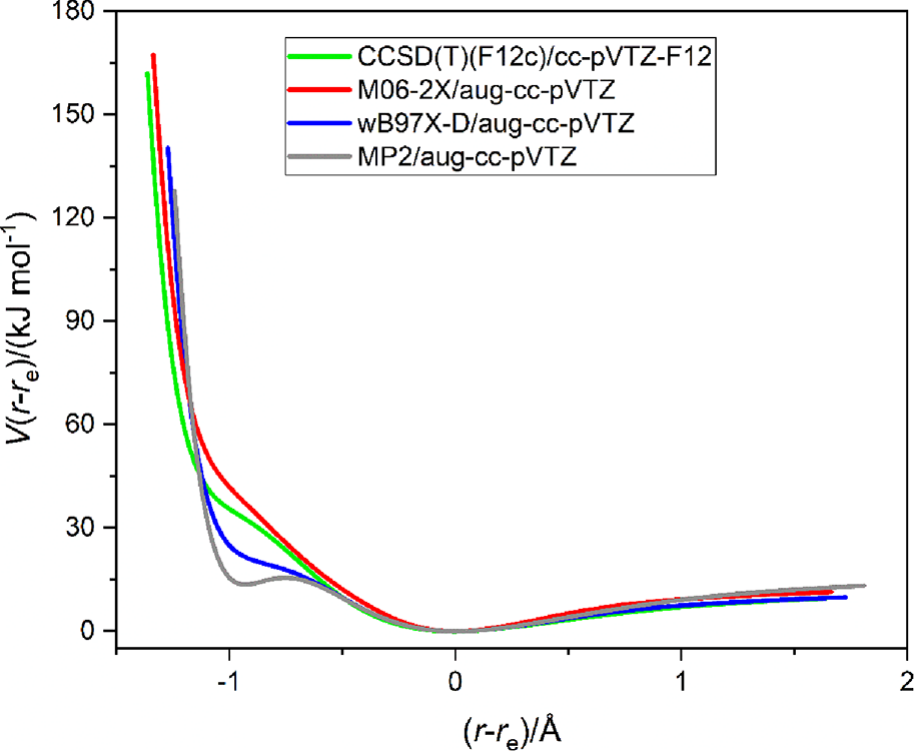
Radial potential energy curves *V*(*r*–*r*_e_) vs (*r*–*r*_e_) of the linear complex OC···ClF calculated at the four indicated levels of theory. Each shows a secondary minimum/inflection at (*r*–*r*_e_) ≈ −1 Å, interpreted to correspond to a geometry to which the valence-bond structure [O=C–Cl]^+^···F^–^ makes a significant contribution. Points were calculated at 0.05 Å intervals and joined by a spline function.

Figure 1 shows the energy *V*(*r*–*r*_e_) calculated as a function of (*r*–*r*_e_), where *r* is the C···Cl internuclear distance and *r*_e_ is its equilibrium value, for the weak, halogen-bonded complex OC···ClF.

The results of four calculations are plotted on the same axes in Figure 1, and, for clarity, the calculated points are not explicitly indicated. Two of the calculations use density functional theory and employ the popular functionals M06-2X and ωB97X-D. The other two calculations were carried out at the MP2/aug-cc-pV(T+d)Z level and the explicitly correlated CCSD(T)-F12c/cc-pVTZ-F12 level. It is clear from Figure 1 that, whatever be the level of theory employed, there is a secondary minimum/point of inflection at (*r*–*r*_e_) ≈ −1 Å, although this appears more pronounced at the MP2 level. The values of *r*_e_ determined by geometry optimization at the four levels of theory were 2.7356, 2.6713, 2.6413, and 2.7604 Å, respectively. An investigation of OC···ClF by rotational spectroscopy concluded that the molecule so observed was a weakly bound,^2^ linear complex, with the atoms in the indicated order and with the distance *r*(C···Cl) = 2.770(3) Å. The experimental value of *r*(C···Cl) was determined under the assumption of unchanged monomer geometries and after allowing for the contribution of the intermolecular bending modes (but not the intermolecular stretching mode) to the zero-point motion. It is the best approximation to the equilibrium value available and is in excellent agreement with that from the CCSD(T)-F12c calculation, thereby confirming that experiment and theory are referring to the same molecular species. The MP2 calculation leads to too short a C···Cl bond (as does ωB97X-D to a lesser extent) and led us to prefer CCSD(T)-F12c and M06-2X calculations in Sections 3.2–3.4. All the four calculations of the one-dimensional PE function indicate that at the secondary minimum/point of inflection, the distance *r*(C–Cl) is in the range 1.70 ± 0.05 Å, which should be compared with *r*(C–Cl) = 1.781 Å for the covalent bond in CH_3_Cl.^29^ Correspondingly, the distances *r*(Cl–F) and *r*(CO) are predicted to be lengthened by 0.16(1) and 0.005(1) Å at the secondary minima/points of inflections. The range of values is that resulting from the average over the calculations at the four levels of theory.

The evidence given in the preceding paragraphs can be interpreted in terms of a simple valence-bond approach. At the secondary minimum/point of inflection, the structure [O=C–Cl]^+^···F^–^ is assumed to make a significant contribution to the valence-bond description of the molecule. Contribution from this structure would result in a molecule with lengthened distances *r*(ClF) and *r*(CO), with the latter change smaller in nature because of the higher bond order, and a significant decrease in the *r*(C–Cl) distance because of the formation of a C–Cl bond.

**Figure 2:**
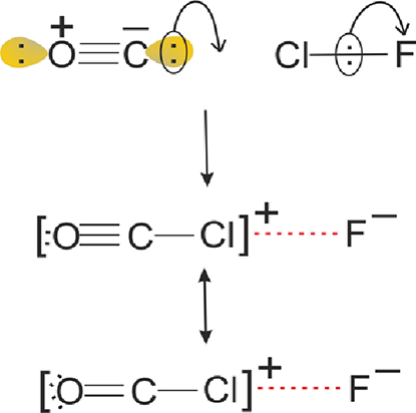
S_N_2-type mechanism for the formation of the geometry found at the secondary minimum/point of inflection in the radial potential energy function of OC···ClF.

The formation of [O=C–Cl]^+^···F^–^ can be envisaged by means of the diagrams shown in Figure 2. According to Pauling,^30^ the predominant valence-bond contribution to the electronic structure of carbon monoxide is that in Figure 2, with both C and O carrying a nonbonding electron pair and (formally at least) the indicated charges. Figure 2 is reminiscent of the S_N_2 mechanism proposed by Ingold.^31^ As OC and ClF approach each other, there is, at a certain distance, a synchronous transfer of the nonbonding pair at C to form the C–Cl bond pair and the transfer of the Cl–F bond pair to F to form F^–^. The double-headed arrow in Figure 2 indicates resonance between two valence-bond structures ascribed to the product. The [O=C–Cl]^+^···F^–^ structure is also consistent with the Mulliken inner complex classification described in Introduction.

### 3.2 Does the Secondary Minimum in the Radial Potential Energy Function of OC···ClF Occur in Other Halogen- and Hydrogen-Bonded Complexes?

The one-dimensional potential energy functions *V*(*r*–*r*_e_) versus (*r*–*r*_e_) of the five axially symmetric complexes N_2_···ClF, OC···ClF, HCN···ClF, H_3_P···ClF, and H_3_N···ClF were calculated in ref (1) at the CCSD(T)-F12c/cc-pVTZ-F12 level. Only the CO complex showed a secondary minimum. The hydrogen-bonded complexes B···HF formed by the same set of Lewis bases with hydrogen fluoride were similarly investigated. None showed the presence of a secondary minimum at small distances (*r*–*r*_e_), perhaps unsurprisingly given that HF has the strongest known single bond and requires much energy to extend it significantly to form [O=C–H]^+^···F^–^.

**Figure 3:**
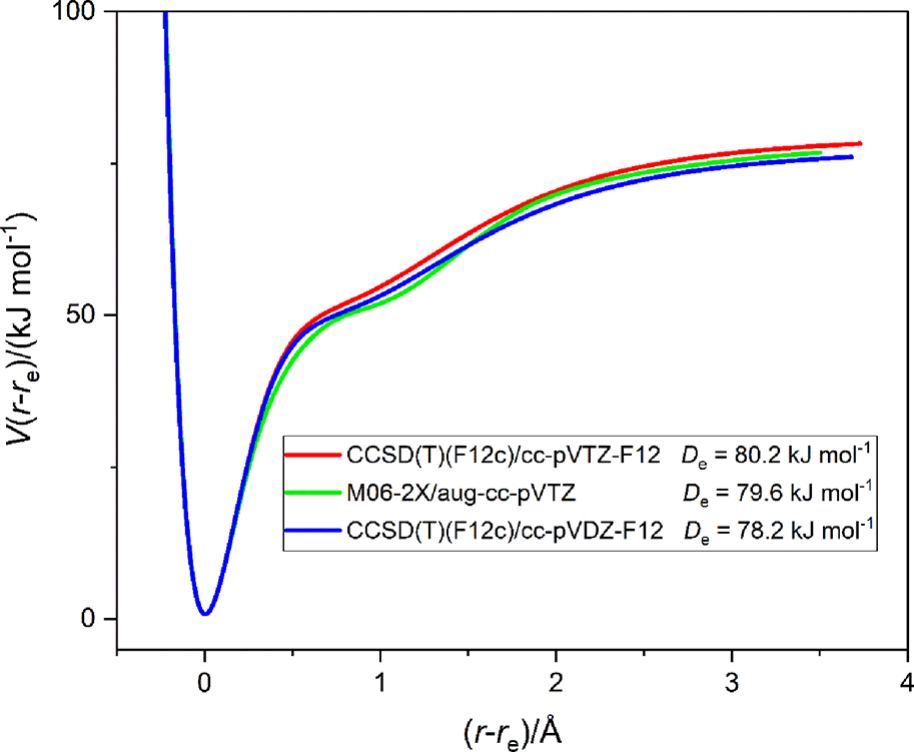
Radial potential energy function *V*(*r*–*r*_e_) vs (*r*–*r*_e_) of SC···ClF calculated at the three indicated levels of theory. Points were calculated at 0.05 Å intervals, with the optimization of other internuclear distances at each point, and were joined by a spline function. Note the close agreement between the calculated dissociation energies *D*_e_. The secondary minima now occur at *r* ≈ 2.6 Å, while the primary minimum is at *r* ≈ 1.61 Å. Note that in this and following figures energies are uncorrected for the basis set superposition error. *D*_e_ in the figures in this article is the energy required to take the complex B···ClF from its hypothetical equilibrium state to infinitely separated components B and ClF, each in its hypothetical equilibrium state.

Perhaps, the molecule carbon monoxide is unique in respect of exhibiting secondary minima of the type [O=C–Cl]^+^···F^–^ in the radial potential energy function of complexes B···ClF. To test this, we calculated this function for SC···ClF, that is, for the halogen-bonded complex in which the chalcogen atom O is replaced by its second-row congener S. The result is shown in Figure 3. Again, the complex was constrained to be linear, and points were calculated at 0.05 Å intervals in (*r*–*r*_e_), with the optimization of *r*(SC) and *r*(ClF) at each point.

Figure 3 shows clearly that there is good agreement between the curves calculated by the DFT method and the explicitly correlated CCSD(T)-F12c method and that there is little difference in the latter case when the basis set is changed from cc-pVDZ-F12 to cc-pVTZ-F12. The values of *r*_e_ for the SC···ClF complex are 1.6111, 1.6195, and 1.6189 Å at the M06-2X/aug-cc-pVTZ and CCSD(T)-F12c/cc-pV*n*Z-F12 (*n* = 2 and 3) levels, respectively. It is striking that although there is, as for OC···ClF, evidence of a secondary minimum, it now occurs at (*r*–*r*_e_) ≈ 1 Å or *r* ≈ 2.6 Å and clearly corresponds to the conventional, weakly bound, halogen-bonded species SC···ClF. The primary minimum, on the other hand, occurs at *r*(C–Cl) = 1.6189 Å (which is very short); the distance *r*(Cl–F) is increased by 0.27 Å from the free ClF value, but the distance between S and C is changed by −0.01 Å from free CS. Thus, the primary minimum now corresponds to an electronic structure, in valence bond terms, that has a significant contribution from the structure [S=C–Cl]^+^···F^–^. The energy required to form infinitely separated CS and ClF from the primary minimum is large at *D*_e_ ≈80 kJ mol^–1^ (uncorrected for BSSE), which is much larger than *D*_e_ = 13.7 kJ mol^–1^ (similarly calculated) for the dissociation process OC···ClF = OC + ClF from its primary minimum. Both CS and CO have the sign of their electric dipole moments μ corresponding to a positive charge on C,^32^ but the magnitude of that of CS is much larger [1.958(5) D]^33^ than the CO value of μ = 0.1222 D.^34^ The greater polarity of CS is likely to lead to an increased preference for the ionic form.

**Figure 4:**
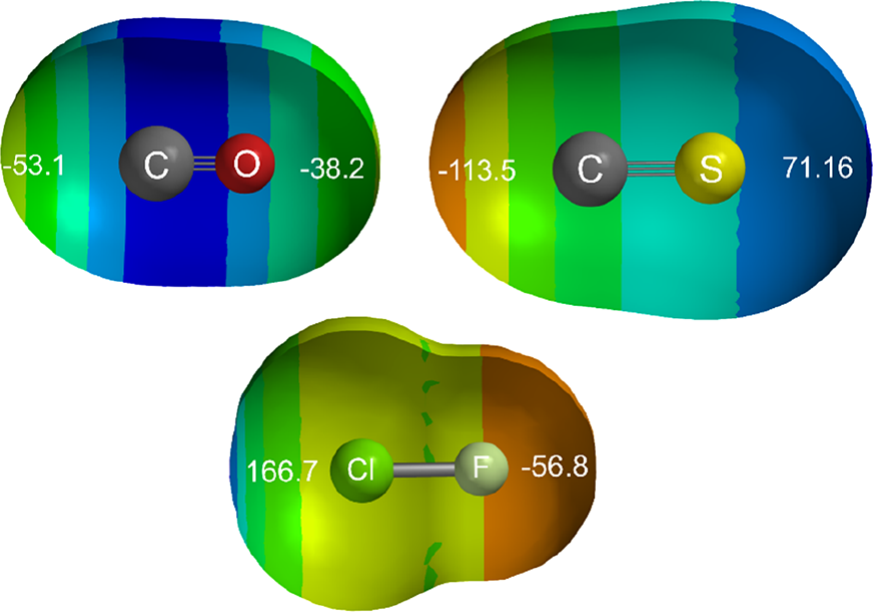
MESPs of carbon monoxide, carbon monosulfide, and chlorine monofluoride calculated at the 0.001 e bohr^–3^ isosurface at the M06-2X/6-311++G** level. Colors at the blue end of the spectrum indicate the more positive (electrophilic) regions of the potential, while those toward the red indicate the more negative (nucleophilic) regions. The numbers in white are in kJ mol^–1^ and indicate the value of the MESP at the isosurface on the molecular axis at each end of each molecule.

The molecular electrostatic surface potentials (MESP) of CO and CS reveal an understanding of the differences in the behavior of these molecules in complexes with ClF. The MESP is commonly defined as the potential energy of a unit charge on the isosurface at which the electron density is 0.001 e bohr^–3^. Figure 4 shows the MESPs at the 0.001 e bohr^–3^ isosurface for CO and CS calculated at the M06-2X/6-311++G** level. Part of the surface has been cut away to reveal the molecular model. We note for CS that the axial region of the isosurface near to C is highly negative (nucleophilic) and likely to undergo a strong interaction with the electrophilic axis region of ClF near to Cl (see Figure 4). The region on the axis near to S is highly electrophilic, however. The situation with CO is quite different. Both of the axial regions of the surface are negative and therefore nucleophilic. Thus, by examining the MESPs of CO and CS, we predict that CO might form two isomeric complexes with the electrophilic region near Cl of ClF (the MESP of which is included in Figure 4), namely OC···ClF and CO···ClF, with the second of these being more weakly bound.

**Figure 5:**
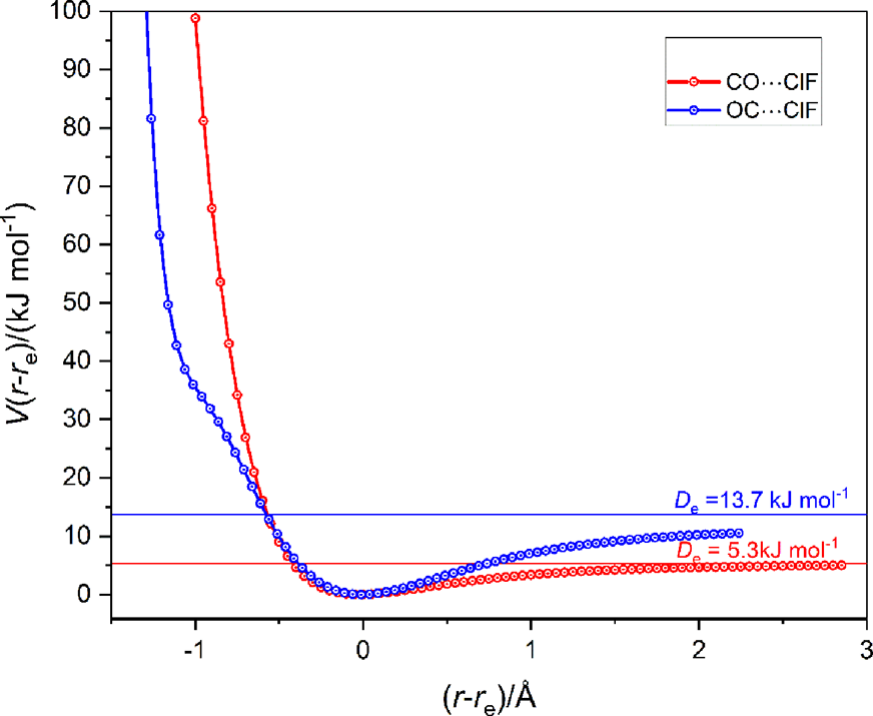
Radial potential energy functions *V*(*r*–*r*_e_) vs (*r*–*r*_e_) of the linear complexes OC···ClF and CO···ClF calculated at 0.05 Å intervals in *r*(X···Cl) (X = C or O) at the CCSD(T)-F12c/cc-pVTZ-F12 level. The points are connected by a spline function.

Displayed in Figure 5 are the radial potential energy functions of the complexes OC···ClF and CO···ClF [calculated at the CCSD(T)-F12c/cc-pVTZ-F12 level in 0.05 Å steps in *r*(X···Cl), X = C or O]. Note that the dissociation energies are consistent with the ratio of the axial values of the MESPs near to C in OC···ClF and O in CO···ClF and that the curve for CO···ClF exhibits no secondary minimum. A similar approach to the SC···ClF and CS···ClF pair is not possible because the calculations at the CCSD(T)-F12c level reveal that CS···ClF is not even weakly bound and has *D*_e_ = −0.1 kJ mol^–1^, a result consistent with the highly electrophilic region of the MESP on the axis near to S.

### 3.3 What Happens if C in the Complexes OC···ClF and SC···ClF is Replaced by its Second-Row Congener Si?

**Figure 6:**
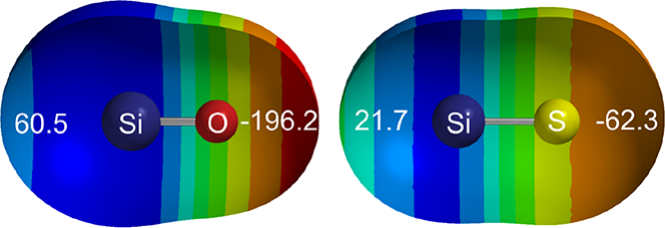
MESPs of silicon monosulfide and silicon monoxide calculated at the 0.001 e bohr^–3^ isosurface at the M06-2X/6-311++G** level. Colors at the blue end of the spectrum indicate the more positive (electrophilic) regions of the potential, while red indicates the more negative (nucleophilic) regions. The values in white on the axes are in kJ mol^–1^ and indicate the values of the MESP on the isosurface and on the axis at each end of each molecule. The deep red region is the most nucleophilic (most negative), while the dark blue region is the most electrophilic (most positive).

The diatomic molecules SiO and SiS (like CO and CS) are well characterized, and all possess^1^Σ^+^ ground states.^35^ The MESPs of SiO and SiS calculated at the M06-2X/6-311++G** level are given in Figure 6. The potentials on the axes near the O and S atoms are both nucleophilic (negative, red), while on the axes near to Si, both regions are electrophilic. Thus, we expect very weak complexes of the type OSi···ClF and SSi···ClF. The question of main interest is: will they nevertheless, like their carbon congeners, show a secondary minimum?

**Figure 7:**
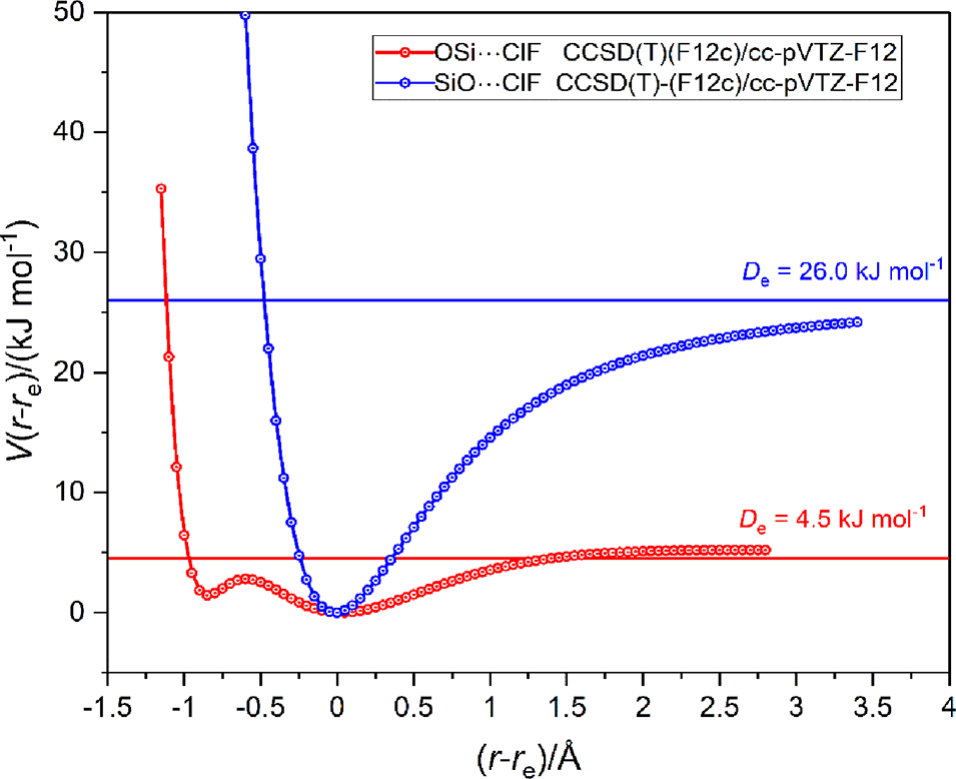
Radial potential energy functions of OSi···ClF and SiO···ClF calculated at 0.05 Å intervals in *r*(X···Cl) (X = Si or O) at the CCSD(T)-F12c/cc-pVTZ-F12 level. The points are connected with a spline function.

**Figure 8:**
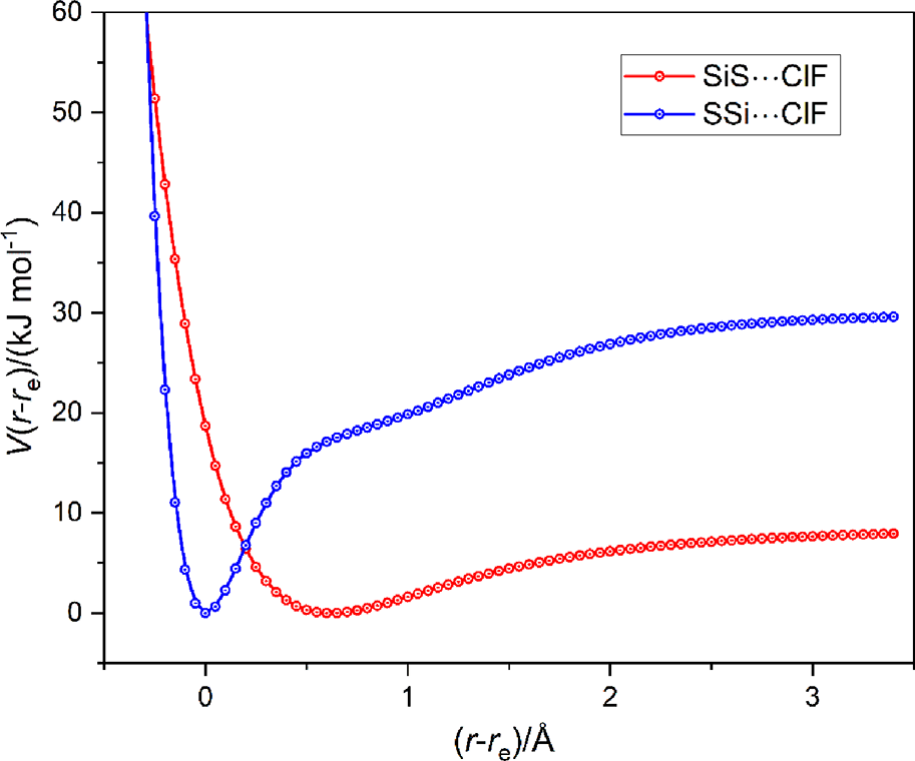
Radial potential energy functions of SSi···ClF and SiS···ClF calculated at 0.05 Å intervals in *r*(X···Cl) (X = Si or S) at the CCSD(T)-F12c/cc-pVTZ-F12 level. The points are connected with a spline function.

Graphs of the radial potential energy functions *V*(*r*–*r*_e_) versus (*r*–*r*_e_) for the two isomers OSi···ClF and SiO···ClF are presented in Figure 7. As previously mentioned, points were calculated at the CCSD(T)-F12c/cc-pVTZ-F12 level at 0.05 Å intervals in the distance *r*(Si···Cl) or *r*(O···Cl), as appropriate, and joined by a spline function. The corresponding diagram for the pair of complexes SSi···ClF and SiS···ClF is presented in Figure 8.

Figure 7 confirms that the behavior of the complexes OSi···ClF and SiO···ClF parallels that of the pair in which Si is replaced by C. Thus, the radial PEF of OSi···ClF has a secondary minimum at approximately (*r*–*r*_e_) = −1 Å, presumably likewise arising from a complex with significant [O=Si–Cl]^+^···F^–^ character. In addition, the complex SiO···ClF_._ has, like its C atom counterpart, only a single minimum in the PE function, the only significant difference being that the dissociation energy *D*_e_ = 26.0 kJ mol^–1^ in the case of the Si complex is larger than *D*_e_ = 4.5 kJ mol^–1^ of the OSi···ClF isomer, while the order is reversed for C in place of Si.

Figure 8 should be compared with Figure 3, which displays the radial PE function for the SC···ClF complex. Recall that CS···ClF was found to be unbound, unsurprisingly in view of the very large positive axial value of the MESP near S. Clearly, the radial PE functions for SC···ClF and SSi···ClF considered here are very similar. Both show a shallow secondary minimum at (*r*–*r*_e_) = 1 and much deeper minima at *r*_e_ = 1.6189 and 1.9347 Å, with *D*_e_ values of 80.2 and 29.6 kJ mol^–1^. Thus, in these cases, the primary minimum also corresponds to a molecule in which the Mulliken inner complex structure [S = T–Cl]^+^···F^–^ (T = a group 14 atom C or Si) makes a substantial contribution to the overall wave function. The value (−62.3 kJ mol^–1^, see Figure 6) of the axial MESP near to the S atom of SiS is negative and therefore nucleophilic, while that near S in CS is positive (71.2 kJ mol^–1^, electrophilic, see Figure 4). Consequently, while CS···ClF is not bound, SiS···ClF is (*D*_e_ = 9.1 kJ mol^–1^). We note again that the radial PE functions of both SiO···ClF and SiS···ClF, in which a chalcogen atom is directly involved in the halogen bond, possess only a single minimum, as is the case for CO···ClF.

The conclusion from the material presented in this section is that the presence of secondary minima in the radial potential energy functions of halogen-bonded complexes formed with ClF as the halogen donor can be predicted from the MESP maps, and, from the complexes investigated, such secondary minima only occur when the halogen bond is formed to one of the group 14 atoms, C or Si.

Possible explanations of the observations made in Sections 3.2 and 3.3 will be advanced in Section 3.5.

### 3.4 Isoelectronic Series FB···ClF, OC···ClF, and N_2_···ClF

The diatomic molecules FB, CO, and NN are isoelectronic, each having at least some triple bond character and a ^1^Σ^+^ ground state.^35^ The radial potential energy functions of OC···ClF and N_2_···ClF calculated at the CCSD(T)-F12c/cc-pVTZ-F12 level have been discussed in an earlier publication.^1^ The function for OC···ClF (as already discussed) has a secondary minimum that can be attributed to the Mulliken inner complex structure of the type [O=C–Cl]^+^···F^–^. Moreover, it was shown that on replacing C by the group 14 second-row atom Si, this behavior persists. In the present section, the effect of moving from OC···ClF along an isoelectronic series to either FB···ClF in one direction along the first row of the periodic table or to N_2_···ClF in the other direction is considered.

**Figure 9:**
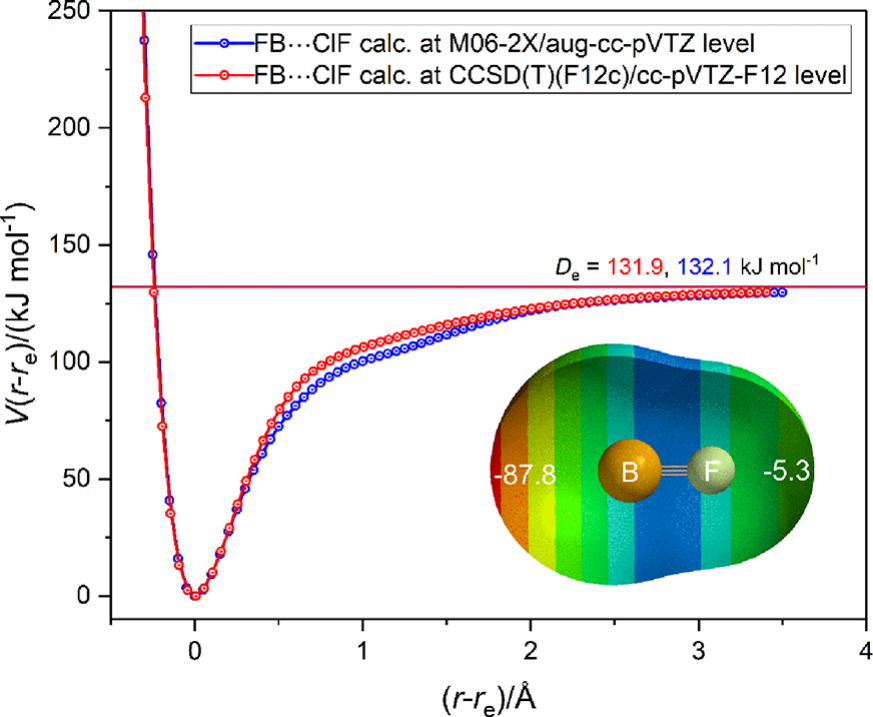
Radial potential energy function *V*(*r*–*r*_e_) vs (*r*–*r*_e_) of FB···ClF calculated at two different levels of theory. Points were calculated at 0.05 Å intervals and joined by a spline function. The inset is the MESP of BF calculated at the M06-2X/6-311++G** level of theory on the 0.001 e bohr^–3^ isosurface. The numbers in white are the values (in kJ mol^–1^) of the MESP at the surface and on the molecular axis.

The radial potential energy of FB···ClF was calculated as a function of the internuclear distance *r*(B···Cl). The energy calculations were conducted at two levels of theory, namely CCSD(T)-F12c/cc-pVTZ-F12 and M06-2X/aug-cc-pV(T+d)Z. For convenience of comparison, the potential energy *V*(*r*–*r*_e_) plotted against (*r*–*r*_e_) for each is displayed in Figure 9. Also shown in Figure 9 is the MESP on the 0.001e bohr^–3^ isosurface for FB calculated at the M06-2X/6-311++G**level. The surface potential on the molecular axis and outside the B atom is large, negative, and therefore likely to be highly nucleophilic.

Both functions in Figure 9 have a very deep primary minimum at *r*_e_ ≈ 1.64 Å, with an equilibrium dissociation energy *D*_e_ = 132 kJ mol^–1^ and the hint of a very shallow secondary minimum at (*r*–*r*_e_) ≈1 Å, and therefore *r* ≈ 2.64 Å.

**Figure 10:**
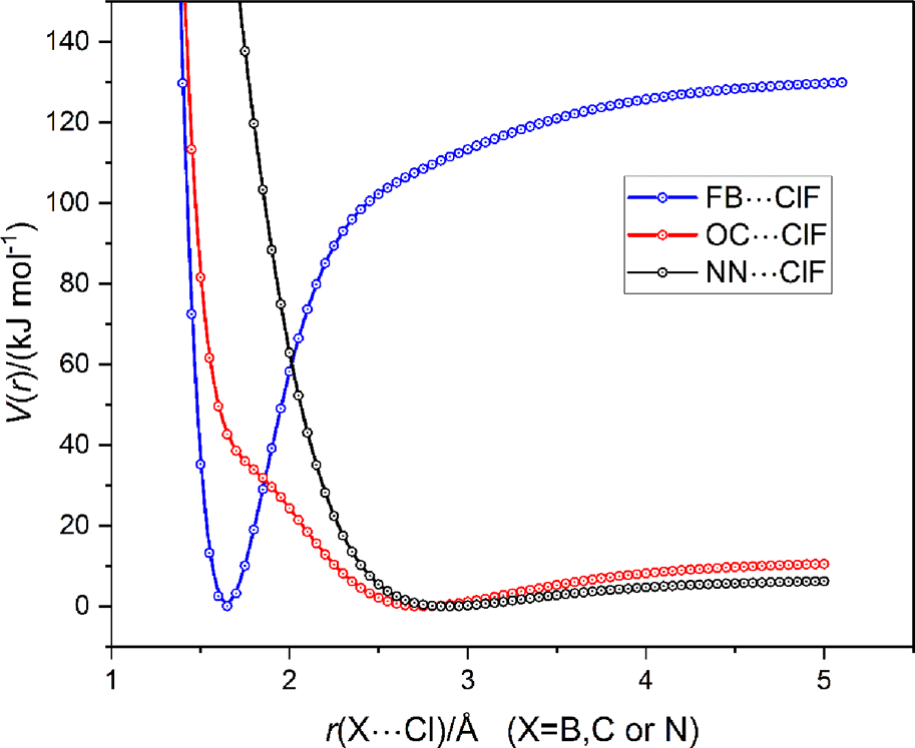
Radial PE curves *V*(*r*) vs *r*(X···Cl) for FB···ClF, OC···ClF, and N_2_···ClF calculated at the CCSD(T)-F12c/cc-pVTZ-F12 level. For the MESPs of BF, CO, and N_2_ on the 0.001 e bohr^–3^ isosurface, as calculated at the M06-2X/6-311++G** level, see Figures 4, 9, and 12, respectively.

Figure 10 shows a plot of *V*(*r*) versus *r*, where *r* is the distance *r*(X···Cl) between the atom X (= B, C, or N) directly adjacent to Cl of ClF in the complexes FB···ClF, OC···ClF, or N_2_···ClF, respectively. This method of presentation shows clearly how much shorter is the equilibrium distance *r*_e_(B···Cl) = 1.6439 Å than those of its counterparts OC···ClF and N_2_···ClF. Moreover, it illustrates that the secondary minimum occurs in the repulsive part of the OC···ClF function but coincides with the primary minimum of FB···ClF. This adds weight to the argument that such minima correspond to molecules in which the Mulliken inner complex structures [F=B–Cl]^+^···F^–^ and [O=C–Cl]^+^···F^–^ make a major contribution. The secondary minimum in the case of the FB···ClF potential energy curve is just detectable and occurs at the same distance *r* as the primary minima of OC···ClF and N_2_···ClF, thereby reinforcing the conclusion that this minimum corresponds to the simple halogen-bonded complex FB···ClF formed first when ClF approaches FB but rapidly destroyed again as the distance *r*(B···Cl) decreases further. On the other hand, it is noted that no secondary minimum occurs in the repulsive part of the N_2_···ClF potential. Evidently, no structure of the type [N=N–Cl]^+^···F^–^ is encountered in the approach of ClF to N_2_. An explanation of why no minima of the Mulliken inner type of complex is observed will be offered in Section 4.

**Figure 11:**
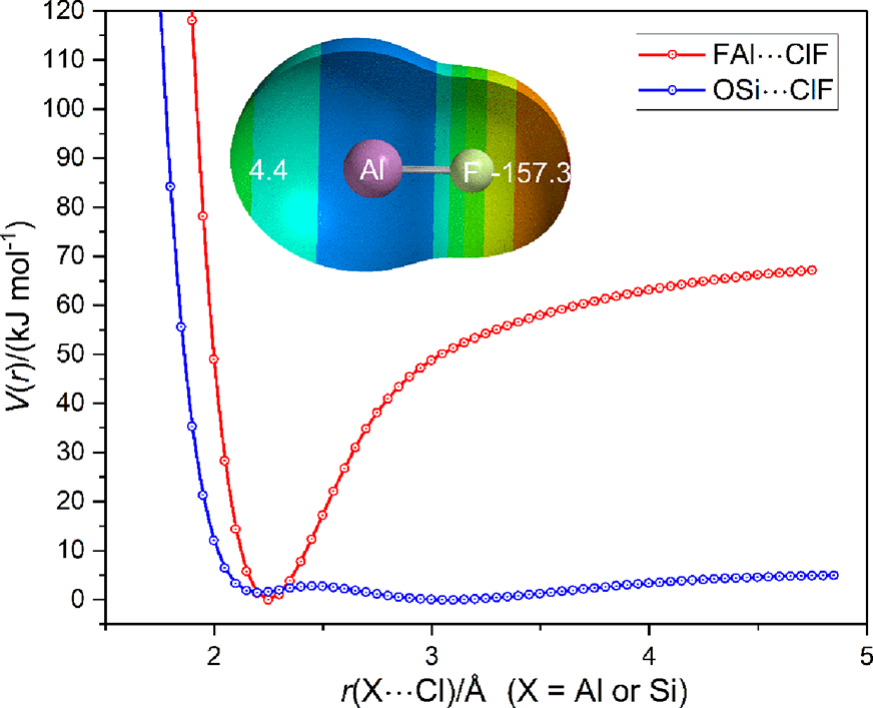
*V*(*r*) vs *r*(X–Cl) for FAl···ClF and OSi···ClF calculated at the CCSD(T)-F12c/cc-pVTZ-F12 level. The primary minima for the two complexes are at *r*_e_ = 2.194 and 3.0480 Å, respectively. The MESP at the 0.001 e bohr^–3^ isosurface of AlF, as calculated at the M06-2X/6-311++G** level, is shown in the inset and can be compared with that similarly calculated for SiO in Figure 6.

It is of interest to compare the counterpart of the FB···ClF, OC···ClF, and N_2_···ClF series in which the atom directly involved in forming the halogen bond with ClF is replaced by the second-row atom of the same group in the periodic table, that is, the series FAl···ClF, OSi···ClF, or NP···ClF. The values of the dissociation energy *D*_e_ are 69.4, 4.5, and 0.3 kJ mol^–1^, respectively (all uncorrected for BSSE), when calculated at the CCSD(T)-F12c/cc-pVTZ-F12 level. Clearly, NP···ClF must be considered unbound, but the radial potential energy functions of FAl···ClF and OSi···ClF calculated at this level of theory can be compared, and these are set out in Figure 11. The relationship of the two potential curves is similar to that observed for FB···ClF and OC···ClF shown in Figure 10.

**Figure 12:**
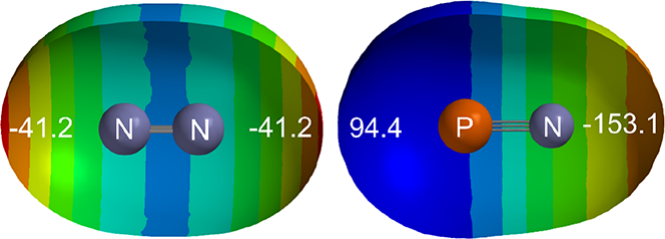
MESPs of N_2_ and PN at their 0.001 e bohr^–3^ isosurfaces. These were calculated at the M06-2X/6-311++G** level of theory using Spartan 20. The numbers in white give the MESP (in kJ mol^–1^) at the point where the molecular axis intersects the isosurface.

It remains to examine the relationship between N_2_ and NP and understand why the complex of the latter with ClF is essentially unbound. Figure 12 displays the MESPs of N_2_ and PN at the 0.001 e bohr^–3^ isosurface in each case, both calculated at the M06-2X/6-311++G* level of theory. The MESP of NP has axial values of 94.4 and −153.1 kJ mol^–1^ at the P and N ends, respectively. Thus, it is clear that the P end of NP is highly electrophilic (positive), and it is therefore not surprising that the complex NP···ClF is essentially unbound when P interacts with the electrophilic Cl end of ClF (see Figure 4 for the MESP of ClF). On the other hand, the N end of NP is highly nucleophilic compared with the corresponding region in N_2_, and therefore the complex PN···ClF is more strongly bound than N_2_···ClF.

**Figure 13:**
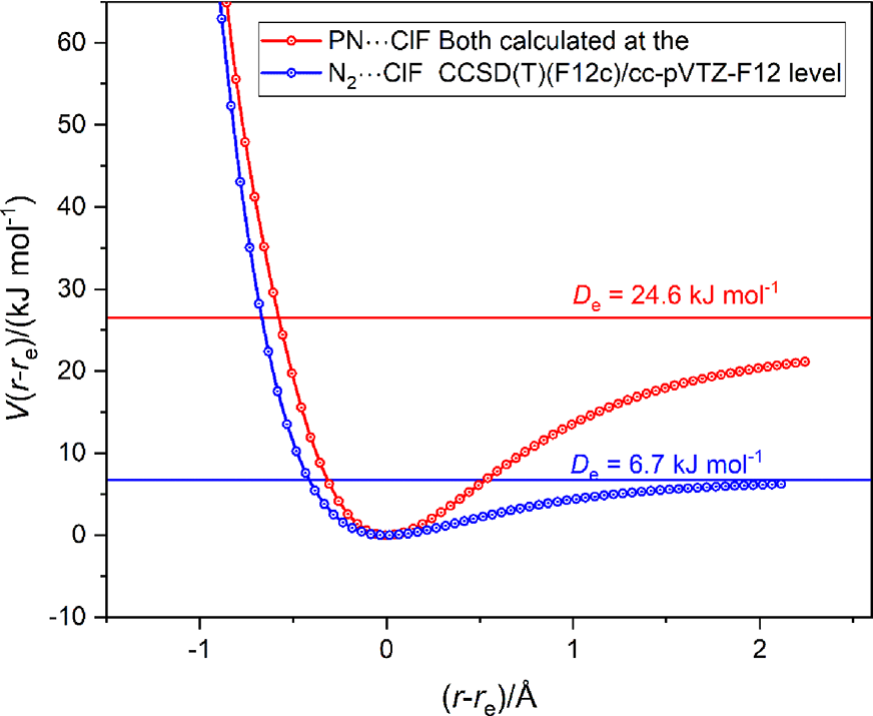
Comparison of the radial PE functions of N_2_···ClF and PN···ClF calculated at the CCSD(T)-F12c/cc-pVTZ-F12 level of theory.

The radial potential energy curves of N_2_···ClF and PN···ClF calculated at the CCSD(T)-F12c/cc-pVTZ-F12 level are displayed in Figure 13. The comparisons in Figures 12 and 13 confirm the conclusion drawn earlier, namely: if the atom Y of a diatomic molecule YX consisting of a pair of first-row atoms is substituted by its second-row analogue, the binding strength of YX···ClF increases. We also note from Figure 13 that, like N_2_···ClF, PN···ClF has only a single minimum in its radial PEF.

### 3.5 SAPT and NBO Analyses of Complexes B···ClF

Further insights into the underlying nature of the interaction in both the primary and secondary minima of the series B···ClF are provided by using SAPT calculations to decompose the interaction energies into a “chemist’s grouping”, with the results given in Tables S1 and S2 of the Supporting Information. Comparisons of the SAPT interaction energies with those from counterpoise-corrected CCSD(T)-F12c/cc-pVTZ-F12 calculations are shown in Tables S3 and S4. In general, there is a very good level of agreement between the two methods, although the level of SAPT chosen does appear to underestimate the strength of the interaction for the most strongly bound complexes. As an explicitly correlated coupled-cluster methodology was used, some of this difference is presumably due to basis set incompleteness errors. The basis set superposition error (BSSE) at the CCSD(T)-F12c/cc-pVTZ-F12 level is also shown in Tables S3 and S4, where the BSSE is typically between 1 and 2 orders of magnitude smaller than the interaction energy, justifying the decision not to include a counterpoise correction in the calculation of the radial potential energy functions.

The SAPT decomposition values of the attractive components of the B···ClF interaction energy are presented as percentages of the total of the attractive terms in Tables 1 and 2, for the primary and secondary minima, respectively. Focusing momentarily on the primary minima, the interaction energies of the complexes SC···ClF, SSi···ClF, and FB···ClF are immediately striking due to their strength. It should be noted that these are interaction energies and hence are missing the energetic effects of distorting the “monomers” from their isolated geometries and are not directly comparable to the analogous dissociation energies presented earlier. Inspecting the contribution of the attractive components within these interaction energies, it is clear that all the three strongly bound complexes have significantly increased charge transfer and reduced dispersion when compared to the primary minima of the other complexes. This supports the designation of Mulliken inner complexes, where structures of the type [B–Cl]^+^···F^–^ would make a significant contribution to the overall valence bond wave function. Table 1 also indicates that the dispersion contribution to the interaction energy is greater than the electrostatic contribution for the primary minimum of complexes CO···ClF and SiS···ClF, and dispersion also makes a large contribution to OSi···ClF and N_2_···ClF.

**Table 1:** SAPT Decomposition of the Attractive Components of the B···ClF Interaction Energy for the Primary Minima as Percentages of the Total of the Attractive Terms

B	electrostatic (%)	induction (%)	dispersion (%)	charge transfer (%)	*E*_I_ (kJ mol^–1^)
OC	47.59	20.12	26.65	5.64	–11.06
CO	37.55	11.51	46.86	4.08	–5.01
SC	45.33	25.75	9.06	19.85	–133.23
SiS	34.28	22.97	37.68	5.07	–8.76
SSi	39.62	27.11	10.45	22.82	–80.76
OSi	28.84	34.71	28.48	7.98	–2.98
SiO	51.28	20.43	21.84	6.45	–26.07
FB	43.00	23.24	8.43	25.32	–211.85
N_2_	43.66	13.64	38.50	4.20	–6.47
PN	50.66	21.75	21.24	6.34	–22.71

**Table 2:** SAPT Decomposition of the Attractive Components of the B···ClF Interaction Energy for the Secondary Minima as Percentages of the Total of the Attractive Terms^a^

B	electrostatic (%)	induction (%)	dispersion (%)	charge transfer (%)	*E*_I_ (kJ mol^–1^)
OC	45.90	26.65	10.90	16.55	+7.86^b^
SC	50.81	20.71	23.19	5.28	–22.05
SSi	35.76	33.09	23.05	8.10	–10.08
OSi	38.24	29.28	11.80	20.68	–28.66
FB	46.43	28.22	18.42	6.93	–22.73

a No secondary minimum was located when CO, SiS, SiO, N_2_, or PN was acting as the Lewis base B.

b SAPT2+(3)(CCD)δMP2/aug-cc-pV(T+d)Z indicates this complex to be unbound.

The SAPT decompositions of the secondary minima shown in Table 2 have a similar pattern; those complexes previously identified as having the Mulliken inner complex character in their secondary minimum, namely OC···ClF and OSi···ClF, have increased charge transfer and reduced dispersion contributions. It should be noted that the OC···ClF secondary minimum has a positive interaction energy, which is consistent with the secondary minimum being in the repulsive part of the radial potential energy curve in Figure 10. Comparison of Tables 1 and 2 reveals that the secondary minimum of OSi···ClF has a stronger interaction energy than the primary minimum. While this seems initially inconsistent with Figure 7, this is again due to the neglect of relaxation energy when considering interaction energy rather than dissociation energy.

**Figure 14:**
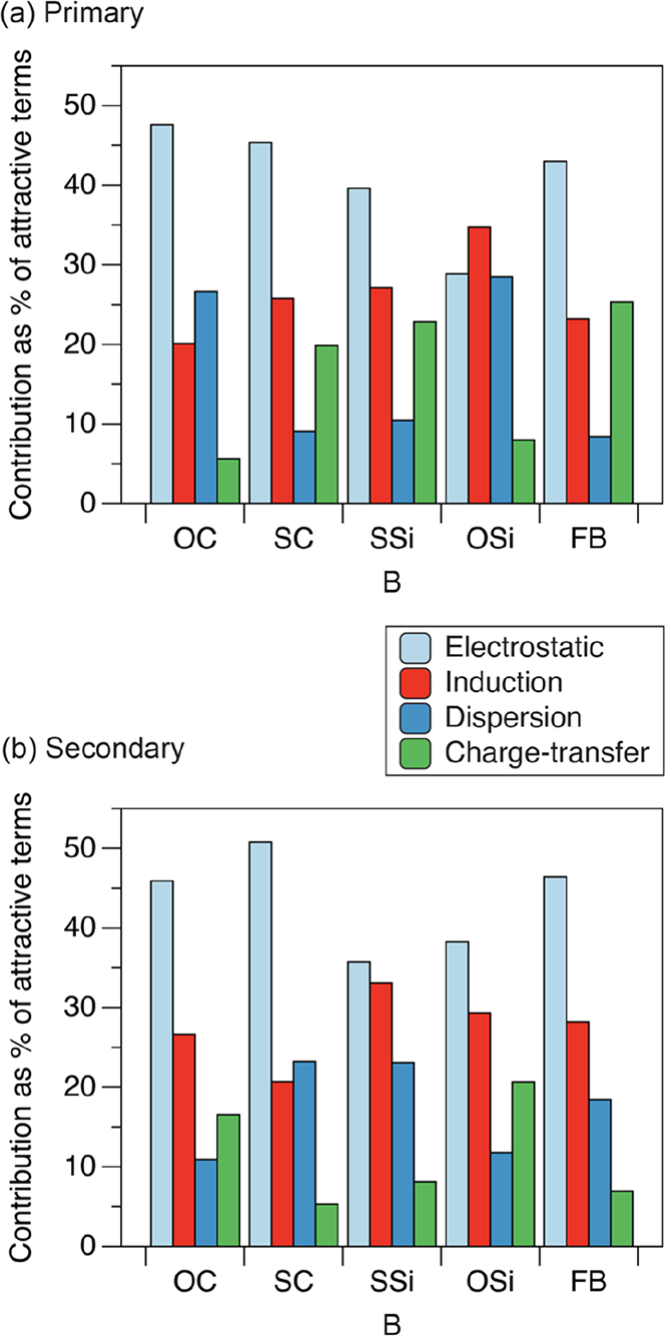
Individual attractive SAPT components of the B···ClF interaction energy as a percentage of the total of the attractive terms. Only the intermolecular complexes found to have both primary and secondary minima are shown.

Figure 14 compares the SAPT components of the interaction energy for those complexes where both primary and secondary minima have been found, showing how the underlying nature of the interaction changes between the two minima. For those complexes with a strongly bound primary minimum, SC···ClF, SSi···ClF, and FB···ClF, the decrease in charge transfer and increase in dispersion on going from the primary to secondary minimum is clearly visible. As expected, OC···ClF and OSi···ClF show this same trend on going from the secondary to the primary minimum. Changes to the other components of the interaction energy are present, but generally less dramatic.

Further evidence for the change in the underlying nature of the interaction in the complexes with both primary and secondary minima can be found from the NBO-derived natural population analysis, summarized in Table 3. The values presented are the partial charges located on the ClF subunit of the complex, with the partial charge on the Lewis base subunit equal in magnitude but opposite in sign (not shown). SC···ClF, SSi···ClF, and FB···ClF show relatively large partial charges for the primary minimum, which are significantly reduced in the respective secondary minimum. Meanwhile, the secondary minimum for OC···ClF and OSi···ClF has a large partial charge, which becomes almost negligible for the primary minimum. This is consistent with the trends in charge transfer from the SAPT analysis above and with those minima of the complexes possessing significant [B–Cl]^+^···F^–^ character (Mulliken inner complexes). For those complexes where no secondary minimum was located, the partial charges are negligibly small in all cases.

**Table 3:** NBO-Derived Natural Population Analysis (NPA) Partial Charges on ClF in the Complex B···ClF^a^

	NPA partial charge on ClF (e)	
B	primary minimum	secondary minimum
OC	–0.03	–0.25
CO	0.00	
SC	–0.15	–0.03
SiS	–0.01	
SSi	–0.55	–0.08
OSi	–0.06	–0.48
SiO	–0.03	
FB	–0.53	–0.09
N_2_	–0.01	
PN	–0.04	

a No secondary minimum was located when CO, SiS, SiO, N_2_, or PN was acting as the Lewis base.

Inspection of the NBO second-order perturbation theory analysis indicates that, for the six minima identified as Mulliken inner complexes above, the electron density is being partitioned as [YX–Cl]^+^···F^–^, with significant intermolecular interactions, where the lone pair on F is donated into an antibonding X–Cl orbital. All remaining minima have the YX···ClF structure with a Cl lone pair donating into an antibonding Y–X orbital, adding further weight to the above classification of Mulliken inner or outer complexes.

## 4 Conclusions

The main conclusions concerning the radial potential energy functions of the YX···ClF complexes considered in this article are conveniently summarized in Table 4. Also included in Table 4 are: the dissociation energy *D*_e_ for the process YX···ClF → YX + ClF, as calculated here at the CCSD(T)-F12c/cc-pVTZ-F12 level; the dissociation energy *D*_e_ of ClF;^36,37^ typical values of Δ*H*(X–Cl) for the dissociation of the X–Cl covalent bond;^38^ the structure from Mulliken’s classification (inner or outer complex); and some comments.

**Table 4:** Summary of Conclusions Concerning the Radial Potential Energy Functions of YX···ClF Complexes

				Mulliken classification structure (inner or outer complex)		
Complex YX···ClF	*D*_e_(XY···ClF)/kJ mol^–1^^a^	*D*_e_(ClF)/kJ mol^–1.^^b^	Δ*H*(X–Cl)/kJ mol^–1.^^c^	primary	secondary	comments
OC···ClF	13.7	257.2	330	OC···ClF	[O=C–Cl]^+^···F^–^	MESP shows CO is axially binucleophilic; strong energy gain by forming C–Cl bond.
CO···ClF	5.3		205	CO···ClF	none	No gain in energy by forming O–Cl covalent bond in CO···ClF
						
SC···ClF	80.2	257.2	330	[SC–Cl]^+^···F^–^	SC···ClF	MESP of CS is strongly nucleophilic at the C end. There is net energy gain for breaking the ClF bond and making C–Cl bond; S end of CS is wholly electrophilic and no significant energy gain by breaking ClF bond and forming S–Cl bond
CS···ClF	∼0		250	unbound	none	
						
OSi···ClF	4.5	257.2	359	OSi···ClF	[O=Si–Cl]^+^···F^–^	MESP of SiO is electrophilic on the axis near Si, so OSi···ClF is weakly bound; net gain in energy by forming Si···Cl bond; strongly nucleophilic at the O end but no energy gain if [SiO–Cl]^+^···F^–^ were to be formed
SiO···ClF	26.0		205	SiO···ClF	none	
						
SSi···ClF	29.6	257.2	359	[S=Si–Cl]^+^···F^–^	SSi···ClF	Si end of SiS is electrophilic but strong energy gain by breaking the ClF bond and forming Si–Cl bond; S end is strongly nucleophilic but no net energy gain by forming the S–Cl bond, hence a single minimum which corresponds to SiS···ClF
SiS···ClF	9.1		250	SSi···ClF	none	
						
FB···ClF	131.9	257.2	494(40)	[F=B–Cl]^+^···F^–^	FB···ClF	Huge energy gain by breaking ClF and making B–Cl or Al–Cl bond; deep minima for the inner complex forms
						
FAl···ClF	69.4	257.2	487(7)	[F=Al–Cl]^+^···F^–^	FAl···ClF	Large energy gain when Al–Cl bond is formed, [FAl–Cl]^+^···F^–^, at primary minimum; secondary minimum at FAl···ClF
						
N_2_···ClF	6.7	257.2	200	NN···ClF	none	N ends of N_2_ and NP are nucleophilic; no energy gain by breaking ClF and making N–Cl bond; no secondary minimum; only minimum is the halogen bond in each case; P end of NP is wholly electrophilic; no energy gain by breaking ClF and making P–Cl bond
PN···ClF	24.6		200	PN···ClF	none	
NP···ClF	unbound		264(40)	none	none	

a Values of *D*_e_ are those displayed in the appropriate figures and are therefore uncorrected for BSSE.

b Data from refs (36) and (37).

c Data from ref (38).

It is assumed that as ClF approaches YX from an infinite *r*(X···Cl) distance, a halogen-bonded system of the type YX···ClF is first encountered at separations of about 3 Å. This corresponds to a minimum in the radial PE curve. As *r*(X···Cl) decreases further, one of two things can happen. First, if the energy required to dissociate ClF into atoms and then to produce the ions Cl^+^ and F^–^ is smaller than the energy gain Δ*E*(X–Cl) through the formation of the X–Cl covalent bond in the ion [Y = X–Cl]^+^, then another minimum in the radial PEF corresponding to a species in which the Mulliken inner complex structure [Y = X–Cl]^+^···F^–^ makes a significant contribution to its electronic structure will be encountered. The larger the energy gain, presumably, the deeper will be this minimum. If, on the other hand, the energy Δ*E*(X–Cl) returned by the formation of the X–Cl bond in [Y = X–Cl]^+^ is insufficient, the Mulliken inner complex structure [Y = X–Cl]^+^···F^–^ will not contribute significantly, and the energy of the system will merely rise as exchange repulsion sets in. Both types of result have been encountered in the investigations reported here. Further evidence for the Mulliken inner or outer complex nature has also been provided by natural population analysis at the minima, and from SAPT decomposition of the interaction energy, which shows clear changes in the underlying nature of the interaction.

Calculation of Δ*E*(X–Cl) requires, inter alia, knowledge of the detailed electric charge distributions of both YX and ClF as well as that in the ion [Y = X–Cl]^+^, ionization potentials, electron affinities, polarization effects, and van der Waals energy and is beyond the scope of the present work. Nevertheless, it is interesting to compare the *D*_e_ values for the process ClF = Cl + F and Δ*H*(X–Cl) for the formation X + Cl = X–Cl of a typical XCl bond. The former is accurately known while a useful compilation of the latter is available.^38^ The appropriate values are included in Table 4. It is immediately obvious from Table 4 that when Δ*H*(X–Cl) is significantly greater than *D*_e_(ClF), the primary minimum in the radial PEF corresponds to a molecule in which the Mulliken inner complex structure is important. When combined with a YX molecule in which the MESP has a large negative value at the X end of the molecule, this leads to very deep minima, as is the case when YX = SC, FB, or FAl. These show weaker minima, corresponding to the simple chlorine-bonded species YX···ClF at larger *r*(X···Cl). When Δ*H*(X–Cl) is closer to *D*_e_(ClF), there can still be two minima, but the depths of the primary and secondary minima are more nearly equal, as is the case for XY = OC, OSi, and SSi. We also note from Table 4 that when Δ*H*(X–Cl) < *D*_e_(ClF), only single minima corresponding to the simple halogen-bonded species XY···ClF are observed. This is true for CO···ClF, SiO···ClF, N_2_···ClF, and PN···ClF, but CS···ClF and NP···ClF are unbound at the level of calculation employed. Thus, it appears that the formation of N–Cl, O–Cl and S–Cl bonds does not provide sufficient energy for the formation of complexes of the type [Y = X–Cl]^+^···F^–^ (X = N, O, S, or P). These are conclusions based on the simple correlation mentioned earlier and must be treated cautiously in view of the neglect of the contributions described. Nevertheless, at that level of approximation, it is concluded that the ion-pair-type minima occur when X = B, Al, C, and Si because of the strength of B–Cl, Al–Cl, C– Cl, and Si–Cl bonds but not for X = N, P, O, and S.

## Supporting Information Available

Grouping of SAPT terms and comparison of interaction energies (PDF)
