# Supplementary material for: Radial Potential Energy Functions of Linear Halogen-Bonded Complexes YX···ClF (YX = FB, OC, SC, N2) and the Effects of Substituting X by Second-Row Analogues: Mulliken Inner and Outer Complexes
Source: J Phys Chem A. 2022 Apr 15;126(16):2511–21. doi: 10.1021/acs.jpca.2c01205 (PMC9097511; doi:10.1021/acs.jpca.2c01205)
Supplement: Supplementary file 1 — jp2c01205_si_001.pdf [file jp2c01205_si_001.pdf]

Supporting Information for “Radial potential energy functions of linear halogen-bonded complexes YX☹ClF, (YX = FB, OC, SC, N_2) and the effects of substituting X by second-row analogues: Mulliken inner and outer complexes.”

J. Grant Hill† and Anthony C. Legon‡
† Department of Chemistry, University of Sheffield, Sheffield S3 7HF, U.K.
‡ School of Chemistry, University of Bristol, Cantock’s Close, Bristol BS8 1TS, U.K

Grouping of SAPT terms

The SAPT2+(3)(CCD)δMP2 truncation within Psi4 was used to decompose the interaction energy. The individual SAPT terms were collected into electrostatic, exchange, induction and dispersion components using the so-called “chemist’s grouping”:

$$E_{\text{electrostatic}} = E_{\text{elst}}^{(10)} + E_{\text{elst,resp}}^{(12)} + E_{\text{elst,resp}}^{(13)}$$

$$E_{\text{exchange}} = E_{\text{exch}}^{(10)} + E_{\text{exch}}^{(11)} + E_{\text{exch}}^{(12)}$$

$$E_{\text{induction}} = E_{\text{ind,resp}}^{(20)} + E_{\text{exch-ind,resp}}^{(20)} + E_{\text{ind}}^{(30)} + E_{\text{exch-ind}}^{(30)} + {}^{t}E_{\text{ind}}^{22} + {}^{t}E_{\text{exch-ind}}^{22} + \delta E_{\text{HF}}^{(3)} + [\delta E_{\text{MP2}}]$$

$$E_{\text{dispersion}} = E_{\text{disp}}^{(20)} + E_{\text{disp}}^{(30)} + E_{\text{disp}}^{(21)} + E_{\text{disp}}^{(22)} + E_{\text{disp-exch}}^{(20)}$$

The SAPT charge-transfer analysis used the SAPT2+(3)(CCD) level and the charge-transfer component was subtracted from the $E_{\text{induction}}$ component. The resulting components for the primary and secondary minima are shown in Tables S1 and S2, respectively.

Table S1. SAPT components (kJ mol^{-1}) of the interaction energies for the primary minima of the complexes B···ClF.

B	Electrostatic	Exchange	Induction	Dispersion	Charge-transfer
OC	–24.23	39.86	–10.24	–13.57	–2.87
CO	–4.86	7.92	–1.49	–6.06	–0.53
SC	–817.43	1669.97	–464.35	–163.42	–358.00
SiS	–11.32	24.27	–7.59	–12.45	–1.67
SSi	–349.62	801.76	–239.27	–92.25	–201.38
OSi	–16.33	53.65	–19.66	–16.13	–4.52
SiO	–42.36	56.55	–16.88	–18.05	–5.33
FB	–823.94	1704.12	–445.30	–161.61	–485.12
N_2	–9.11	14.39	–2.85	–8.03	–0.88
PN	–50.35	76.70	–21.63	–21.12	–6.31

Table S2. SAPT components (kJ mol^{-1}) of the interaction energies for the secondary minima of the complexes B···ClF. No secondary minimum was located when CO, SiS, SiO, N_2 or PN were acting as the Lewis base.

B	Electrostatic	Exchange	Induction	Dispersion	Charge-transfer
OC	–420.39	923.64	–244.06	–99.80	–151.52
SC	–32.66	42.23	–13.31	–14.91	–3.40
SSi	–31.36	77.61	–29.02	–20.21	–7.10
OSi	–240.81	601.06	–184.36	–74.31	–130.23
FB	–42.68	69.18	–25.94	–16.93	–6.37

Comparison of interaction energies

The total SAPT2+(3)(CCD)δMP2/aug-cc-pV(T+d)Z interaction energies are compared to those at the counterpoise corrected (CP) CCSD(T)-F12c/cc-pVTZ-F12 level for the primary and secondary minima in Tables S3 and S4, respectively.

Table S3. Comparison of the total SAPT2+(3)(CCD)δMP2/aug-cc-pV(T+d)Z (SAPT) and counterpoise corrected CCSD(T)-F12c/cc-pVTZ-F12 [CP-CCSD(T)-F12c] interaction energies for the primary minima of the complexes B···ClF. The magnitude of the basis set superposition error (BSSE) at the CCSD(T)-F12c/cc-pVTZ-F12 level of theory is also shown.

	Interaction energy (kJ mol^{-1})		
B	SAPT	CP-CCSD(T)-F12c	BSSE (kJ mol^{-1})
OC	–11.06	–11.53	0.24
CO	–5.01	–5.13	0.16
SC	–133.23	–138.09	1.70
SiS	–8.76	–8.92	0.26
SSi	–80.76	–77.97	0.96
OSi	–2.98	–4.37	0.28
SiO	–26.07	–26.06	0.35
FB	–211.85	–220.29	1.57
N_2	–6.47	–6.79	0.18
PN	–22.71	–24.94	0.35

Table S4. Comparison of the total SAPT2+(3)(CCD)δMP2/aug-cc-pV(T+d)Z (SAPT) and counterpoise corrected CCSD(T)-F12c/cc-pVTZ-F12 [CP-CCSD(T)-F12c] interaction energies for the secondary minima of the complexes B···ClF. The magnitude of the basis set superposition error (BSSE) at the CCSD(T)-F12c/cc-pVTZ-F12 level of theory is also shown. No secondary minimum was located when CO, SiS, SiO, N_2 or PN were acting as the Lewis base.

	Interaction energy (kJ mol^{-1})		
B	SAPT	CP-CCSD(T)-F12c	BSSE (kJ mol^{-1})
OC†	7.86	3.62	1.15
SC	–22.05	–22.61	0.26
SSi	–10.08	–11.01	0.35
OSi	–28.66	–28.43	0.72
FB	–22.73	–22.77	0.29

†Both SAPT and CCSD(T)-F12c interaction energies indicate this complex is unbound.
